# A Review of Silica-Based Nanoplatforms for Anticancer Cargo Delivery

**DOI:** 10.3390/ijms26125850

**Published:** 2025-06-18

**Authors:** Andrea Mosseri, Leticia Sanchez-Uriel, Jose I. Garcia-Peiro, Felipe Hornos, Jose L. Hueso

**Affiliations:** 1 Instituto de Nanociencia y Materiales de Aragón (INMA), CSIC-Universidad de Zaragoza, Campus Río Ebro, Edificio I+D, C/Poeta Mariano Esquillor, s/n, 50018 Zaragoza, Spain; amosseri@unizar.es (A.M.); lsuriel@unizar.es (L.S.-U.); joseignacio.garcia@unizar.es (J.I.G.-P.); 2 Department of Chemical and Environmental Engineering, University of Zaragoza, Campus Río Ebro, C/María de Luna, 3, 50018 Zaragoza, Spain; 3 Networking Research Center in Biomaterials, Bioengineering and Nanomedicine (CIBER-BBN), Instituto de Salud Carlos III, 28029 Madrid, Spain; 4 Instituto de Investigación Sanitaria (IIS) de Aragón, Avenida San Juan Bosco, 13, 50009 Zaragoza, Spain; 5 Instituto de Investigación, Desarrollo e Innovación en Biotecnología Sanitaria de Elche (IDiBE), Universidad Miguel Hernández, 03202 Elche, Spain; 6 Escuela Politécnica Superior, Universidad de Zaragoza, Crta. de Cuarte s/n, 22071 Huesca, Spain

**Keywords:** silica, mesoporous, stimuli responsive, tumor microenvironment, enzyme, metals, cancer therapy, drug delivery, oligonucleotides

## Abstract

Stimuli-responsive silica nanoparticles have emerged as a promising platform for the targeted and controlled delivery of therapeutic agents in cancer therapy. These nanoparticles possess unique physicochemical properties that allow for the stimuli-triggered release of loaded cargos, such as drugs, enzymes, oligonucleotides, photosensitizers, and metals. The stimuli-responsive nature of these nanoparticles enables them to respond to specific internal and external signals within the tumor microenvironment, including pH, temperature, and redox potential, among others. This leads to the enhanced targeting of cancer cells and improved therapeutic efficacy while minimizing the off-target effects. This review highlights recent advances in the development and application of stimuli-responsive silica nanoparticles for the delivery of multiple active agents for cancer therapy. Overall, stimuli-responsive silica nanoparticles offer great potential for the development of more effective cancer therapies with improved selectivity and reduced side effects.

## 1. Introduction

Over the past few decades, porous materials have shown immense potential for providing sustainable solutions to various global issues, such as increasing energy demands, improving industrial pollutant standards, resource depletion, and better health outcomes [1,2]. Porous particles can drive physical and chemical processes by exposing surface atoms to different local environments as compared to those in the bulk, resulting in a higher specific surface area and increased material reactivity, leading to improved efficacy in various applications [2,3,4]. Based on the pore size, porous particles can be classified into three groups: microporous (less than 2 nm), mesoporous (between 2 and 50 nm), and macroporous (greater than 50 nm) [3]. Mesoporous particles, with their unique structural properties, such as a long-range ordered pore structure and uniform pore size, have been extensively studied and utilized in various fields, such as catalysis, adsorption, separation, sensing, and biomedical applications [3,4,5,6,7,8]. Mesoporous silica nanoparticles (MSNs) are by far the most studied material, with applications such as enzyme immobilization. Other interesting examples include mesoporous carbon nanoparticles (MCNs), metal–organic frameworks (MOFs), and zeolites [3].

Drug delivery systems (DDSs) based on nanoparticles (NPs) are designed to specifically deliver therapeutic agents to solid tumors, increasing the effectiveness of anticancer treatment while minimizing their systemic toxicity [9,10]. Since the 1980s, the enhanced permeability and retention effect (EPR) has been considered the main mechanism enabling the entry and accumulation of NPs in tumors [11,12]. This preferential accumulation of therapeutic agents has driven cancer nanomedicine research. However, its limited clinical translation has revealed that the EPR effect is insufficient to explain the delivery of NPs to solid tumors. Consequently, alternative mechanisms have already been described, such as the active transport and retention (ATR) principle [13], which proposes that NPs enter tumors via active transport processes, and not only passively through inter-endothelial gaps. This perspective suggests that the mechanisms of NP delivery are more complex than previously anticipated. Furthermore, in 2016, a comprehensive analysis by Wilhelm et al. [9] revealed that only 0.7% of systemically administered NPs reach tumor sites. When administered intravenously (IV), NPs face several biological barriers from the injection site to the site of action, including the adsorption of serum opsonin proteins onto their surfaces, interactions between NPs and the immune system, the selective extravasation of NPs at tumor sites, the penetration of NPs into solid tumors, and the internalization of NPs by tumor cells [13,14,15,16]. Overcoming these biological barriers is crucial for the eradication of tumor cells, which require efficient NP entry into cancer cells and the ability to penetrate the dense tumor extracellular matrix and high interstitial fluid pressure of tumor tissue [15,16].

Silica (SiO_2_)-based nanoplatforms, in particular MSNs, hold great promise as carriers for loading, delivery, and in vivo cancer therapy applications, due to their controlled porosity and their functionalization capabilities. Chen et al. [17] corroborated that 25 nm-sized PEGylated and doxorubicin (DOX)-loaded MSNs exhibited a superior ability to penetrate the blood–brain barrier (BBB) and evidenced the in vivo inhibition of glioma growth, accumulating abundantly in the tumor. A biodistribution analysis of tumor-bearing mice also showed that when there was low or no PEG, MSNs were mainly trapped in the liver. The same MSNs were then used in another work [18] to study their behavior in a model of the chorioallantoic membrane (CAM), an extraembryonic membrane crucial in the embryonic development of oviparous animals. The carcinoma-bearing CAM showed a pattern of biodistribution and accumulation similar to that of the mouse model.

**Scheme 1:**
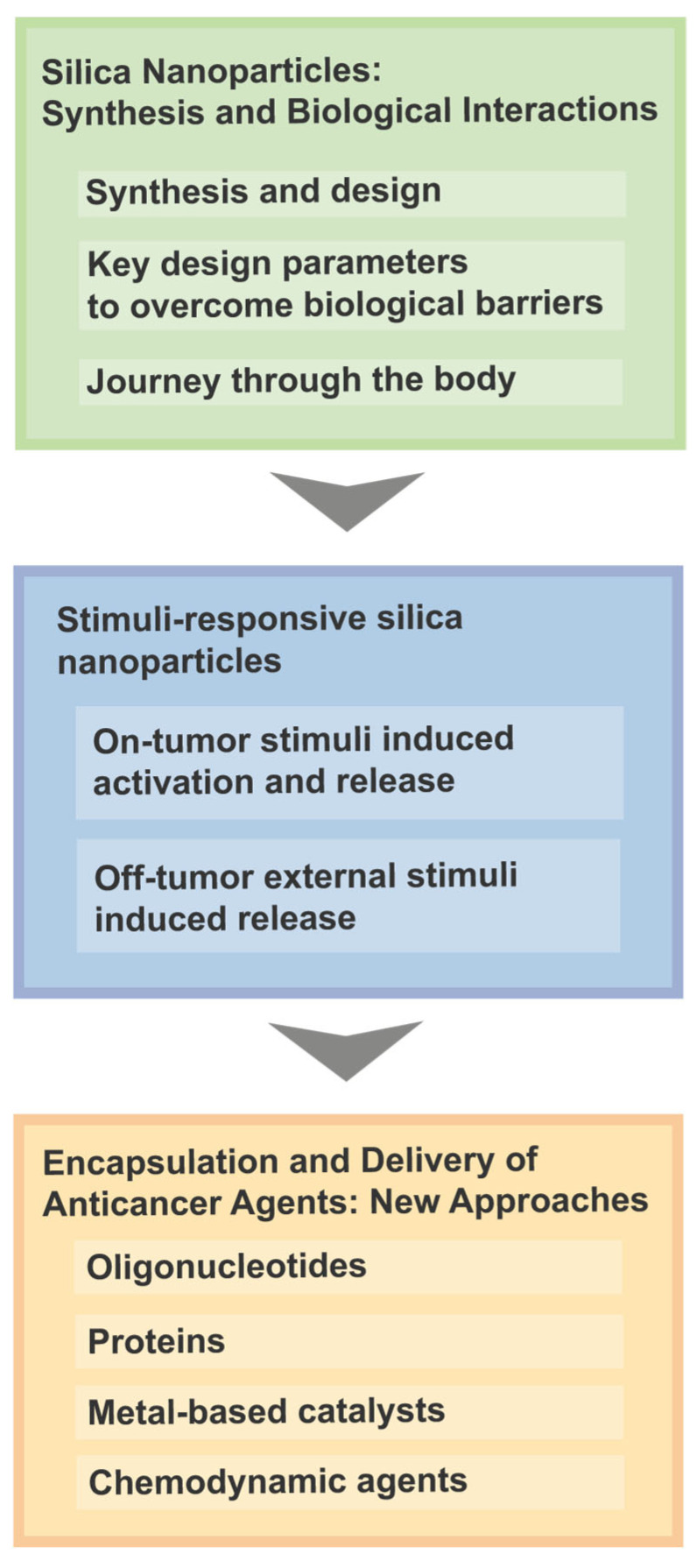
Flowchart of the main sections and topics covered in this review.

The present review surveys the latest advances in the use and performance of silica-based nanoparticles (SNPs), which have been designed and developed to improve the therapeutic and diagnostic efficacy index, reduce the off-target toxicity, and adjust the pharmacokinetic profile [3,19,20]. These nanosystems have exhibited stimuli-responsive release and active targeting properties (achieved through ligands like antibodies, aptamers, and peptides) [4,19,21,22,23,24,25,26]. To help readers navigate through the main sections and topics covered in this review, we have included a concise flowchart in Scheme 1 (vide infra).

## 2. Silica Nanoparticles: Synthesis and Biological Interactions

### 2.1. Synthesis and Design

**Figure 1:**
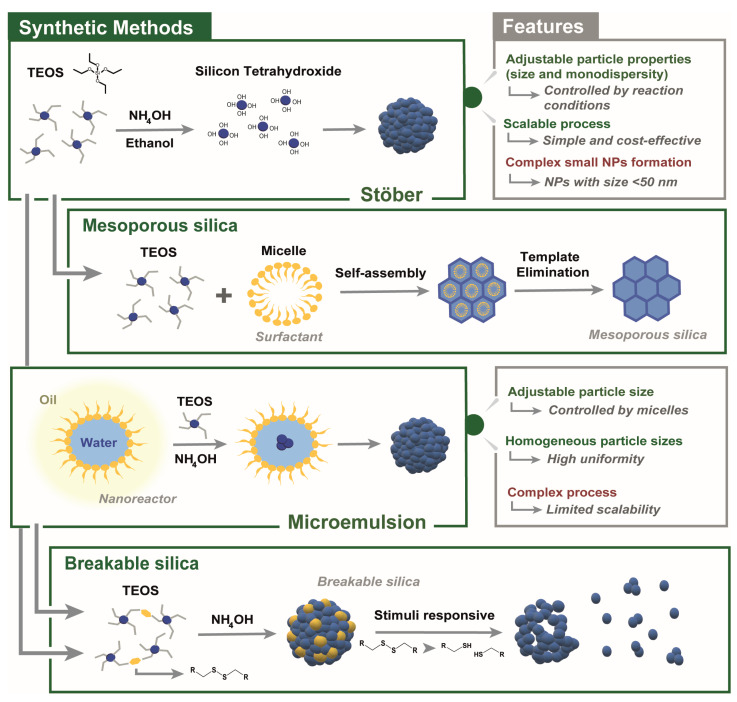
Scheme of different silica nanoparticles synthetic pathways with corresponding features.

There are multiple and well-established strategies for the synthesis of non-porous organosilica NPs [27]. Spherical SNPs are usually obtained by using the reverse microemulsion or the Stöber method (Figure 1) [28]. The Stöber method can be used to create silica particles that are relatively uniform in size [28,29], ranging from 30 nm to 2 μm in diameter. This process involves the hydrolysis and condensation of siloxane precursors, such as tetraethylorthosilicate (TEOS), in the presence of ethanol and ammonia [29]. In this case, organic dye molecules can be also incorporated into the silica particles by covalent attachment through a two-step process: the dye is bound to an amine-containing silane agent, such as 3-aminopropyltriethoxysilane (APTS), and then APTS and TEOS are hydrolyzed and co-condensed to form dye-doped particles [30,31,32,33]. These NPs present great potential for fluorescence bioimaging applications [28], although they exhibit fewer advantages than their mesoporous counterparts do.

Due to their homogeneous structure, large pore volume, and high surface area [1,20,27,34], most studies nowadays take advantage of the ease with which MSNs can be produced (Figure 1). MSN synthesis is carried out by using surfactant-templated co-condensation [35,36] or by reacting diglyceroxysilane in water or buffer solutions without ethanol or methanol [37]. After the formation of the NPs, an extra layer of linker molecules with reactive functional groups, such as amine, thiol, carboxyl, or methacrylate, is frequently added. This is accomplished by applying an additional silica coating (post-grafting) that contains the desired functional group(s) [38]. The functional groups serve as the reaction sites for bioconjugation and alter the colloidal stability of NPs in solutions. For instance, inert negatively charged organosilane compounds containing phosphonate groups can be incorporated into NPs during post-grafting, which enhances the repulsive forces between the particles in a solution, leading to improved long-term NP stability. Mobil Crystalline Materials (MCMs)-41 and MCMs-48, and Santa Barbara Amorphous (SBA)-15 and SBA-16, are types of MSNs possessing different morphologies and pore size ranges [4,8,39].

A reverse micelle or water-in-oil (w/o) microemulsion system (Figure 1) is a homogeneous mixture of oil, water, and surfactant molecules that are isotropic and thermodynamically stable [28,40,41]. Within this system, water nanodroplets form in the oil phase and act as confined nanoreactors for the formation of discrete particles [42,43,44]. This methodology allows for the trapping and encapsulation of polar and water-soluble fluorescent dye molecules, due to the electrostatic attraction between the dye and the negatively charged silica shell matrix [28,45,46,47]. This can be performed by introducing a hydrophobic silica precursor or using water-soluble dextran-conjugated dyes under acidic conditions [40,45]. A microemulsion system has the advantage of yielding highly uniform and monodisperse NPs [28,48], but there is a risk of fluorophores detaching from the silica matrix over time [47]. Additionally, extensive washing is required to remove surfactant molecules before any biological application to prevent their disruption or lysis of biological membranes. Uniform silica coatings as thin as 1 nm have been created for the encapsulation of ferromagnetic NPs via microemulsion [46], and APTS has been added onto the surface of preformed SNPs to generate highly fluorescent monodisperse double-layer SNPs [40].

The Stöber and microemulsion synthesis methods can be tuned to incorporate cleavable organic moieties within the silica frameworks under specific stimuli (Figure 1) [39,41,49,50,51]. The synthesis protocols that follow a modified sol-gel method introduce the organosilica precursor in the ethanol phase, subsequently mixing it with the aqueous phase containing the surfactant [49,50,51,52]. When the double microemulsion approach is used instead, the organosilica precursors are dissolved in the aqueous phase, undergoing polymerization inside the water core of the micelles together with the silica precursors (e.g., TEOS) [41,53,54,55,56,57,58]. The organosilica precursors are composed of a silane extremity (usually ethoxy–methoxy silane) bonded through an alkyl chain to a cleavable moiety. The silica framework is now enriched in cleavable organic functional groups, able to be broken under certain conditions, producing the so-called stimuli-responsive SNPs [39,41,49,51,52,59].

### 2.2. Key Design Parameters to Overcome Biological Barriers

**Figure 2:**
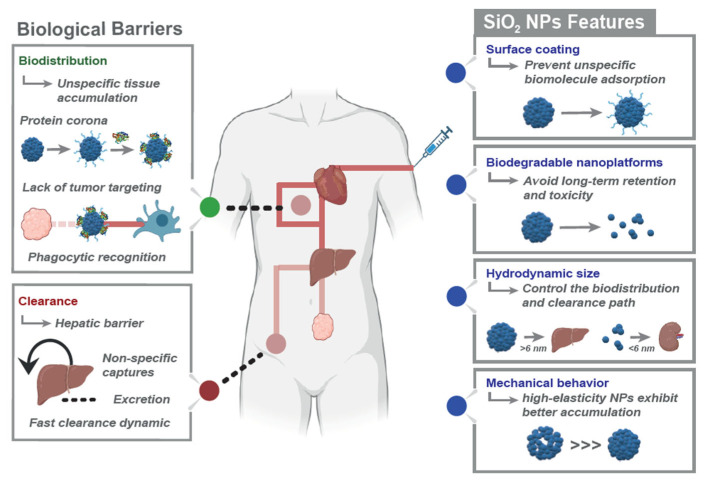
Silica nanoparticles and their journey through body. Scheme describing silica nanomaterials biodistribution and clearance and particular features.

The design of SNPs must be adjusted prior to their administration and interaction with physiological media. Contact with biological fluids, such as blood, causes changes in the NPs at the physicochemical level [60], regardless of how carefully the NPs have been designed or how effective the NPs have been in in vitro assays. A low targeting efficacy or reduced specificity for targeted organs or tumors [9] reinforce the importance of protecting NPs from undesired interactions that can lead to aggregation and early detection by phagocytic cells [60,61]. For instance, polyethylene glycol (PEG) [13,60], a hydrophilic polymer, is commonly conjugated to the surface of NPs (known as PEGylation [62]) to decrease non-specific binding by preventing the adsorption of unwanted charged biomolecules. As a result, the circulating half-life of NPs is increased and the uptake by undesired cells in cultures and mice in vivo is decreased. Moreover, the choice of the type of PEG molecules, as well as their ratio, is determinant for MSNs to be able to cross the BBB both in vitro and in vivo, and to successfully target a tumor [17]. Accordingly, NPs must be designed taking different features into account (see Figure 2).

#### 2.2.1. Influence of Particle Size

Depending on their size, SNPs can enhance their retention and toxicity in different organs of the body (Figure 2). In vitro and in vivo studies have highlighted the toxicity and mechanisms of SNPs on pulmonary cells and tissues. In vitro studies have showed that smaller SNPs (e.g., 7–10 nm) induce pro-inflammatory responses to a higher extent, including oxidative stress and tight junction disruption in bronchial epithelial cells (BEAS-2B) and lung fibroblasts, via reactive oxygen species (ROS)-mediated pathways [63]. Small SNPs can easily access the lung tissue and broncho-alveolar fluid, causing acute lung inflammation, fibrosis, alveoli damage, and other effects [63]. Regarding the neural system, apparently SNPs sub-50 nm in size can exhibit size-dependent toxicity. Various examples in the literature have shown how small silica particles can easily cross the BBB, causing oxidative stress in neural stem cells and astrocytes [64,65,66,67], while not being internalized in neurons. Yang et al. [66] demonstrated that 15 nm SNPs promoted the accumulation of β-amyloid 1–42 and the phosphorylation of tau, both of which are key pathological markers of Alzheimer’s disease (AD). Apparently, a low dose of 100 nm SNPs was safe to use in neurons and stem cells [68]. Their liver toxicity has been widely studied because of the importance of this organ in terms of the metabolism and accumulation of NPs [17,18]. Small SNPs around 15–20 nm can cause oxidative stress, glutathione (GSH) depletion, and apoptosis in liver cells [66,69,70,71]. Damage to hepatic cells is correlated with the p53, Bax/Bcl-2, and caspase-3 pathways. Different administration routes have been evaluated, independently of size, and all the SNPs exhibited liver toxicity in experimental animals causing fibrosis [15,60,72,73,74]. All these examples clearly suggest the need to establish new synthesis routes that ensure the complete biodegradation and excretion of SNPS, as will be further discussed (vide infra).

#### 2.2.2. Influence of Shape and Mechanical Properties

Their shape can affect their circulation mobility and consequently the contact between the particles and vessels/cells membrane. Most studies have focused on spherical NPs, not considering how different shapes of viruses and bacteria can avoid immune system recognition, enhancing their in vivo circulation. The geometry of SNPs significantly influences their hemolytic activity, molecular margination, and cellular uptake. Spherical SNPs, with their low aspect ratio, exhibit higher hemolytic activity due to their larger surface area and small curvature, which makes the hemolysis process more thermodynamically favorable. In contrast, geometries with higher aspect ratios show reduced hemolytic capacity, particularly at lower concentrations [75]. Additionally, cellular uptake varies by NP shape, as rod-shaped NPs are internalized more efficiently, while cube-shaped NPs demonstrate lower internalization rates [75]. Not only the external geometry, but the porosity and size of the pores regulate their interaction with plasma proteins, influencing the circulation half-life and the toxicity of the SNPs [63,75,76,77]. Larger pores show far less thrombogenic activity in comparison to a small (20 nm) pore size [26,78].

The mechanical properties also seem to play a crucial role in biodistribution, targeted delivery, and cellular uptake (Figure 2). Deformable (soft) NPs promote prolonged circulation and reduced accumulation in the spleen. The theoretical models have predicted that “soft” materials are less prone to wrapping by the plasma membrane, making “stiff” materials more energetically favorable for cellular uptake [73]. Hui and co-workers [72] studied in more detail the effects of NPs’ elasticity on receptor-mediated cellular interactions [79]. The group developed a library of SNPs with Young’s moduli ranging from 0.56 kPa to 1.18 GPa, and investigated their interactions with macrophages and cancer cells, confirming how the elasticity can affect macrophage phagocytosis and receptor-mediated uptake by cells, with stiff nanomaterials being preferentially internalized. However, the study also pointed out how cancer cells can lose their stiffness sensitivity during non-specific chlatrin/caveolin independent endocytosis [72].

#### 2.2.3. Influence of Surface Chemistry: Charge, Coating, Stealth, and Cloaking

Another relevant parameter to consider in cellular uptake is the surface charge of the nanomaterial. Positively charged carriers are readily internalized by cells due to the negative charge of the phospholipids in the membranes and, on the other hand, are easily recognized and excreted by macrophages [80]. Moreover, the surface charge is a relevant parameter when considering the interaction of silica NPs with serum proteins. Souris et al. reported that cationic SNPs exhibited a higher propensity to bind to serum proteins, leading to their rapid excretion via the hepatobiliary pathway in vivo compared to anionic SNPs [81]. Clemments et al. and other groups have shown that the surface charge of MSNs strongly affects protein corona formation [76,82]. Vallet-Regi’s group also tested different functional groups, including zwitterionic configurations, which elicited different responses [83]. Cationic amino-functionalized MSNs exposed to 10% fetal bovine serum (FBS) adsorbed the widest variety and highest number of proteins in the hard corona compared to bare or carboxylic acid-functionalized MSNs. Thanks to their surface potential, negatively charged SNPs exhibited longer circulation times but faced challenges in passing through membranes due to electrostatic repulsion [84,85]. Synthesizing charged-reversal particles [86,87] could be one successful approach to overcome the potential issues related to this. Li et al. [88] demonstrated how coating SNPs with functional groups prone to changing their surface charge based on the pH could produce negatively charged circulating NPs that can switch to positive under the acidic conditions found in a tumor microenvironment (TME).

Given the importance of bio-interface interactions [89], additional efforts beyond PEGylation have exploited the targeting capabilities of other functional biomolecules, such as antibodies, aptamers, and peptides [90,91]. Efforts are currently being devoted to exploring biomimetic cloaking designs, which mimic the designs from nature [89,90,92]. A cell has an extremely complex and functional fundamental and unique design. Biological membranes offer a promising strategy for coating silica NPs, enhancing critical parameters to improve therapeutic efficacy [93]. Unlike synthetic coatings, membrane-coated NPs can actively enhance the biodistribution profile of chemotherapeutics and improve tumor penetration and homology-driven targeting [94]. Cell membrane-coated nanoparticles (CNPs) have arisen from this, combining the properties of synthetic NP cores with the bio-interfacing properties of cell membranes [89,90]. MSNs were synthesized as rod-shaped coated membranes from colorectal cancer cells (HT-29 colon adenocarcinoma cell line) [95]. The membranes were obtained by density gradient centrifugation. The superior penetration of rod-shaped MSNs through the colorectal mucosa compared to that of spherical and non-membrane-coated MSNs was obtained in vitro.

Extracellular vesicles (EVs) hold significant potential as drug delivery systems, both independently and in conjunction with artificial NPs. Their inherent properties, such as their ability to overcome biological barriers and migrate toward specific tissues, make them attractive for targeted therapies [96,97,98,99,100]. Bioinspired nanocarriers, based on MSNs coated with EVs, combine the advantages of both components, offering high drug loading and release capabilities, while the EVs provide native homing properties toward their parent cells [101,102,103]. These hybrid nanosystems can selectively kill specific cancer cells without harming healthy cells. In an interesting work by Dumontel et al. [93], they loaded DOX into MSNs due to its fluorescence properties, which allowed for DOX intracellular tracking and release. A significant increase in the mean intensities of HGUE-GB-39 and HeLa cells related to DOX red fluorescence was observed when the cells were treated with MSNs@DOX–EVsGB and MSNs@DOX–EVsHeLa, respectively. Exploiting a different approach, Yong et al. [104] demonstrated how a porous silica material loaded with DOX (DOX@PSiNPs) could be effectively accumulated in tumoral cells and cancer stem cells. In that work, they generated exosome-coated DOX@PSiNPs (DOX@E-PSiNPs) by incubating NPs in cancer cells and waiting for exocyted-coated nanomaterials to develop. After intravenous injection, the DOX@E-PSiNPs showed improved tumor accumulation, deeper tumor penetration, and efficient uptake by both bulk cancer cells and cancer stem cells (CSCs).

Red blood cell (RBC) membranes, cancer cell membranes, macrophage membranes, and platelet membranes have also been reported to improve the circulation lifetime, biocompatibility, immune evasion, and tumor targeting [105,106]. RBC membrane-coated MSNs have been developed to mimic natural blood cells, preventing immune clearance and enabling tumor-specific drug release. For instance, Shao et al. designed RBC-coated MSNs carrying chlorin e6 and DOX, demonstrating a prolonged circulation time and improved photodynamic therapy. Similarly, cancer cell membrane-coated MSNs have leveraged homology recognition to enhance tumor targeting [107]. This strategy was used to efficiently deliver therapeutic cargoes, such as chemotherapeutics [108] or sonosensitizers [109], and prevent macrophage recognition. Despite their advantages, biomimetic MSNs face challenges, such as reduced structural flexibility. Further research is needed to optimize their mechanical properties, large-scale production, and long-term safety for clinical applications [110].

### 2.3. Journey Through the Body

Another important aspect that needs to be addressed for SNPs’ clinical translation is related to the formation of a “protein corona” after an IV injection of NPs, due to their circulation in the bloodstream where they are in contact with serum proteins [13,60,61,82] (Figure 2). This corona interacts with different cells and tissues in the NPs’ pathway to a tumor [111], facilitating binding to the membrane receptors that call for cellular uptake. This phenomenon can suppress active targeting and cause abnormal biodistribution, unexpected toxicity, and low theragnostic efficacy. Then, NPs are mainly cleared from circulation through renal clearance and the mononuclear phagocytic system (MPS) (Figure 2), which consists of phagocytic cells that filter the blood to eliminate particles from circulation [60].

Recent studies have claimed that spherical NPs with a hydrodynamic diameter (HD) below 6 nm can cross through the glomerular capillary walls in the kidneys, being excreted into the urine [60]. This filtration is dependent on the previously mentioned parameters (vide supra, Figure 2). For larger sizes, there are a plethora of recent studies on the biodistribution of NPs that have illustrated their elevated accumulation in the liver compared to other organs [13,17,60,61]. This phenomenon seems to take place regardless of the size, shape, surface coating (even with PEGylated NPs [17]), and chemical composition of the NPs and animal model [60,82]. Liver macrophages, also known as Kupffer cells, are professional phagocytes responsible for such rapid and non-specific capture. The elimination of macrophages and active phagocytic cells by phosphonates [60] has been described as preventing the capture of NPs in Kupffer cells. However, the effects could be more detrimental than beneficial. Strategies aimed at impeding the ability of liver macrophages to interact with NPs are more promising, for example, by decorating the NPs with “do not/eat me” signals [112]. As mentioned before, a “stealth layer” can also be created on the surface of NPs by grafting hydrophilic polymers or macromolecules [113] to improve stability, prevent unspecific biomolecule adsorption, and inhibit immune cell interactions. Although this strategy may not ensure that the NPs reach a tumor, it may help to reduce their premature clearance from circulation and a lead to a higher probability of overcoming the hepatic barrier [60,61,113,114,115].

If the nanosystems successfully circumvented sequestration by the MPS; overcame other barriers, such as opsonization and intratumoral pressure gradients; and performed their therapeutic activity on the target, the particle debris should completely leave the body by biodegradation and/or excretion (Figure 2) to avoid long-term retention and the risk of severe toxicity. Biodegradable NPs and renal-clearable NPs have been approved for clinical trials. Non-degradable nanomaterials pose biosafety concerns and have hardly been approved by regulatory agencies [113]. Silica can degrade slowly in aqueous media due to the hydrolysis of the -Si-O-Si- bonds into two -Si-OH units [116,117]. In particular, the nucleophilic interaction between the hydroxyl groups in aqueous media and the non-bridging oxygen on the surface of MSNs generates soluble silicic acid [21,117,118]. The degradation behavior depends on factors, such as the degree of condensation of the structure, particle size, pore size and texture, degree of aggregation, functionalization groups, and the presence of inorganic or organic species in the silica structure [117,118].

The in vivo degradation of MSN-based nanosystems with an HD larger than 8 nm still have to deal with the relatively long-term bioaccumulation [21,118]. However, metal ions doping (such as iron, manganese, and calcium), cleavable bonds’ covalent incorporation, and silica skeleton reconstructing (Si-O-R) [116] have recently been considered as effective strategies to improve MSNs’ biodegradability [117]. A study conducted by Tang and co-workers [119] on the toxicity and clearance of rattle-type hollow MSNs (HMSNs) without any modification to ICR (Institute of Cancer Research) mice, found that the lethal dose 50 (LD50) of HMSNs was higher than 1000 mg kg^−1^ for the single-dose toxicity, and no deaths were observed when the mice were injected with HMSNs at 20, 40, and 80 mg kg^−1^ by continuous IV administration over 14 days. However, the continuous intraperitoneal (IP) injection of these HMSNs increased liver injury markers in the serum and induced silicotic nodular-like lesions in the liver in a dose-dependent manner. The study also found that HMSNs mainly accumulated in mononuclear phagocytic cells in the liver and spleen, and their entire clearance time required more than 4 weeks [116].

## 3. Stimuli-Responsive Silica Nanoparticles

**Figure 3:**
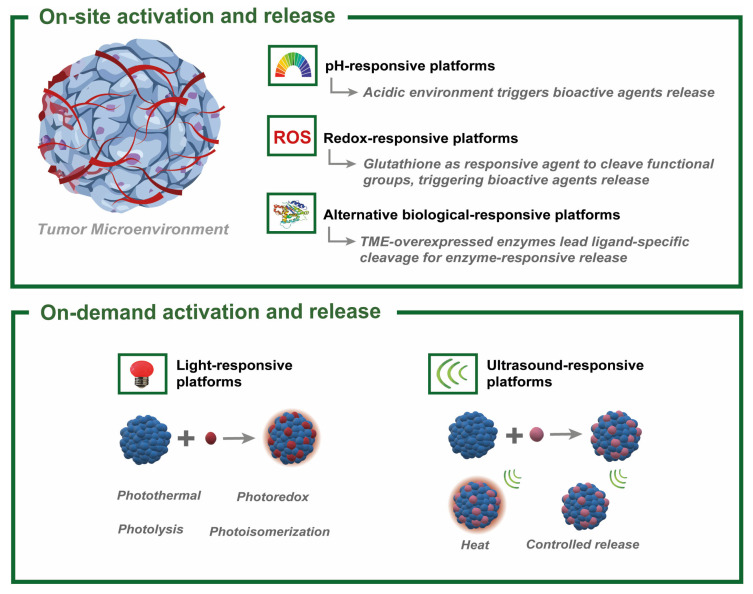
Scheme of silica nanoparticles responding to different stimuli to perform reactive release.

The systemic administration of nanomedicines in high concentrations to overcome biological barriers and to ensure they reach the tumor may seem logical. However, this approach often causes adverse side effects in healthy tissues and organs. In this regard, the possibility that the therapy may only exert its action in response to specific disease stimuli [23] or the TME (Figure 3) has been raised. Recent studies revealing their successful arrival and accumulation in a tumor [17,18], while avoiding the MPS, could lead to promising results for this approach. This would ensure that the SNPs are activated and act only once the NPs reach the tumor. Silica-based nanomaterials containing a cleavable silica framework have acted as stimuli-responsive DDSs [41,49,50,51,52,54,55,59,120], which hold great promise for minimizing side effects and improving the selectivity of treatments. The stimuli to which these systems respond can be physical (temperature, light, ultrasound, magnetism, and electrical stimuli), chemical (pH and oxidative state), or biological (enzymes and metabolites) [4]. Herein, we highlight some of the most successful examples recently explored in the literature, distinguishing between on-site activation and release induced within TMEs and the off-tumor external stimuli used to activate cargo release.

### 3.1. On-Tumor Stimuli-Induced Activation and Release

#### 3.1.1. pH

**Figure 4:**
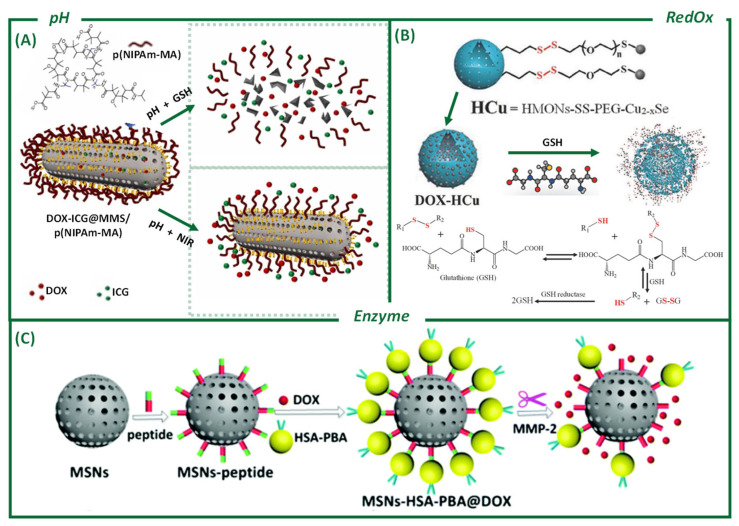
Different examples from the literature of endogenous stimuli-responsive mesoporous silica nanoparticles for anticancer agent release. (**A**) pH: The antitumor drug DOX and photosensitizer ICG are adsorbed into the Mn-doped mesoporous nanorod pores and p(NIPAm-MA) is conjugated on the carrier surface to block the pores. The synthesized DOX-ICG@MMS/p(NIPAm-MA) displays a pH/light-responsive release. Reprinted with permission from Elsevier (reproduced from Chen, M. et al. [122]). (**B**) RedOx: Functionalization and cargo loading using mesoporous NPs with a disulfide-bridged silsesquioxane framework, and the further GSH-triggered degradation along with cargo release. Copyright permission from Wiley-VCH Verlag GmbH & Co. KGaA, Weinheim (reproduced from Du, X. et al. [130]). (**C**) Enzyme: Enzyme MMP-2-responsive-MSNs DDS for targeted tumor therapy in vitro and in vivo. The MSNs’ functionalization with the polypeptide consists of two components: the cell-penetrating peptide polyarginine and the MMP-2 cleavable substrate peptide (PVGLIG). PBA (Phenylboronic acid)–HAS (human serum albumin) is used as the end-capping agent for sealing the mesopores and immobilizing them onto the MSNs via the intermediate linker of polypeptides. Reprinted with permission from The Royal Society of Chemistry (reproduced with permission from Liu, L. [134]).

pH plays a vital role in biological systems. Its physiological value is 7.4 (blood circulation and healthy tissues) and is relatively low in cellular organelles, such as lysosomes (5.5–4.5) [4]. In the case of a TME, its intracellular value is 7.0–7.2, while the extracellular is acidic. Using the pH to manipulate the behavior of functionalized MSNs is nowadays a very promising strategy for the development of antitumor drugs [4] and DDSs based on silica. Controlled drug release can be triggered by changes in the pH of the environment. Manganese (Mn)-doped MSNs have showed almost complete degradation after 48 h in an acidic (pH 5.4) and reducing environment (such as a TME) [121]. The Mn-O bond is easily broken under these conditions, accelerating the degradation of the particle. In contrast, MSNs without metal doping have remained unchanged [117], corroborating the metal doping of MSNs as a strategy to facilitate their biodegradation. In another work by Chen et al. [122], combined photo-chemotherapy and pH- and light-sensitive release were achieved. Mn-doped mesoporous silica nanorods were prepared with a high specific surface area, allowing for the adequate adsorption of the antitumor drug DOX and indocyanine green (ICG) photosensitizer [122] (Figure 4A). Calcium could be also used as a cleavable moiety, as presented in the work of He et al. [123], where calcium cations (Ca^2+^) and disulfide bonds were used as breakable linkers in the silica framework. Ca^2+^ produces sensitivity to changes in the pH, while the disulfide bond is reduced under redox conditions. The dual response permits the release of the drug under TME conditions.

A relevant strategy for designing pH-based drug release from a silica structure is the introduction of Schiff bases. Imine and azomethine groups drive the possibility of cleaving SNPs shells under acidic conditions (as in TMEs). One of the most famous imine examples was presented in the work of Travaglini et al. [49], in which imine silane was previously synthesized and then used, together with TEOS, as the silica source. Liu et al. synthesized a Schiff base-bridged silane precursor to dope a mesoporous silica structure and release DOX under a tumor’s acidic pH [124]. pH-based degradation and release is possible not only by doping the silica framework. “Pore-blocking” is a valid strategy to avoid the leaching and release of a drug from a porous silica material before it reaches the TME. Bis-silylated pyridine-bridged diurea derivative [125] was used to block the release of 5-fluorouracil and ibuprofen from MSNs. Small metal NPs can also be exploited to prevent drug release by grafting mesoporous large pores onto NPs. Inorganic NPs, such as copper sulfide (CuS) [126] and iron oxide (FeO_x_) [127], dissolve in an acidic pH and open the “gates” of the pores, releasing the anticancer drugs.

#### 3.1.2. Reduction–Oxidation Processes (RedOx)

In an analogous way, the development of redox-sensitive platforms is another effective approach for precise and controlled drug delivery. TMEs are characterized by a very high concentration of an antioxidant agent that helps cancer cells to counteract oxidative stress: GSH. Its intracellular concentration is 2–10 mM, and it is 2–10 μM outside cells. This difference allows for the release of drugs into tumor cells [128]. GSH can cleave redox-cleavable groups, triggering the release of bioactive agents. Several studies have taken advantage of these redox imbalance characteristics, demonstrating the efficacy of these nanostructures at delivering chemotherapeutic agents with high specificity and minimal off-target effects (Figure 4B). For instance, Hadipour Moghaddam et al. [110] designed hollow MSNs with a high loading capacity (9%, DOX) and glutathione-sensitive mechanism, allowing for controlled drug release in response to intracellular GSH levels, thereby improving the therapeutic outcomes, with a selective toxicity for RAW 264.7 macrophages and NIH 3T3 cell lines. Similarly, Wang et al. [128] developed single-hole, glutathione-responsive, degradable hollow silica NPs, further enhancing the biodegradability and stability of the drug-loaded carriers, with a loading capacity of 14% (DOX) and the efficient growth inhibition of TCA8113 cells.

A dual-response DDS, to pH and redox, was designed using an amide reaction [129]. NPs of MSN–sulfur–chitosan allowed for the release of salicylic acid, achieving in vitro a 23% release in the presence of GSH, with a significant increase as the pH decreased. Alternatively, the release kinetics of the cargo from mesoporous silica species can also be controlled by hindering disulfides with various surface engineering strategies. For instance, the release rate of loaded cargos from MSNs was successfully regulated by hindering the disulfide-linking β-cyclodextrin nanocaps on the surface of the silica [130]. Redox-responsive disulfide or tetrasulfide groups have also been incorporated into silica frameworks to control delivery upon NP degradation (Figure 4B) [131]. This approach has been used to deliver small- and large-sized cargoes, such as anticancer drugs and proteins, respectively [26,76,77].

#### 3.1.3. Enzymes and Other Biological Stimuli

Using enzymes as the triggers for controlled release has advantages, such as high specificity and negligible adverse effects [4]. MSNs and organosilica NPs can also be utilized for controlled drug release via the enzymatic cleavage of ester, peptide, urea, and oxamide bonds [132]. To achieve this, an ester bond is formed between the stalk and the adamantine stopper, which allows porcine liver esterase to trigger the release of the cargo in a controlled manner [133].

A biocompatible and enzyme-responsive DDS based on MSNs as DOX drug containers was designed. In detail, silica was functionalized with an intermediate ligand and a substrate of a specific enzyme. The enzyme, a metalloproteinase (MMP-2), was overexpressed in the TME. Therefore, when the system reached the tumor, the enzyme would break that ligand, thereby exposing the DOX drug directly to the cancer cells. The system demonstrated a good curative effect via tumor growth inhibition with minimal toxic side effects [134]. De La Torre et al. [135] described a gated-MSNs enzyme-responsive targeted delivery system. The SNP pores were blocked thanks to the T22 peptide grafted onto the surface. This peptide not only directed the SNPs specifically to the CXCR4 receptor of B cell non-Hodgkin’s lymphoma, but was also sensitive to the proteases present in the cancer cells, opening the pore gates only in the TME. In another recent study [136], DOX-loaded MSNs were attached to a self-immolative gatekeeper, which reacted with the high concentration of nitroreductase (NTR) in hypoxic tumor cells.

Another biological stimulus to which MSNs may respond is adenosine-5-triphosphate (ATP), as it is the main source of energy in all living organisms and is the key metabolite in energy-intensive pathological processes, such as tumor progression. In addition, glucose-sensitive nanomaterials are another example, as glucose is the main nutrient in tumor cells [4]. Martinez-Mañez’s group described a pore-capped MSN in which the release of insulin was determined by the opening of the pores under glucose stimulus [137]. The formation of inclusion complexes between cyclodextrine–glucose oxidase and benzoimidazole groups grafted onto the silica surface drove the formation of a glucose-sensitive gatekeeper. In the presence of a high concentration of glucose, the pores of the MSNs opened, and the fluorescein isothiocyanate (FITC)-labeled insulin was then released.

Moreover, several articles have also focused on the development of novel silica-based NPs with controllable cargo release under relevant TME conditions. Yang et al. [138] explored dendritic mesoporous organosilica NPs with structure-dependent biodegradability, optimizing their performance for safe and efficient protein delivery. Ribonuclease A, a potent cytotoxic enzyme, was encapsulated in dendritic mesoporous silica with a tunable pore size. The smaller pore-sized NPs enabled a more controlled release profile compared with larger ones, resulting in the better accommodation of proteins within pores. PBA (Phenylboronic acid)–HAS (human serum albumin) was used as the end-capping agent for sealing the mesopores and immobilizing them onto the MSNs via the intermediate linker of polypeptides (Figure 4C) [134]. Employing enzyme/biomolecule reactive groups to dope the silica framework is an effective strategy, due to the high presence of these molecules in TMEs. Exploiting the key metabolites overexpressed in TMEs is a successful approach for selectively targeting cancer cells.

### 3.2. Off-Tumor External Stimuli-Induced Release

#### 3.2.1. Light

**Figure 5:**
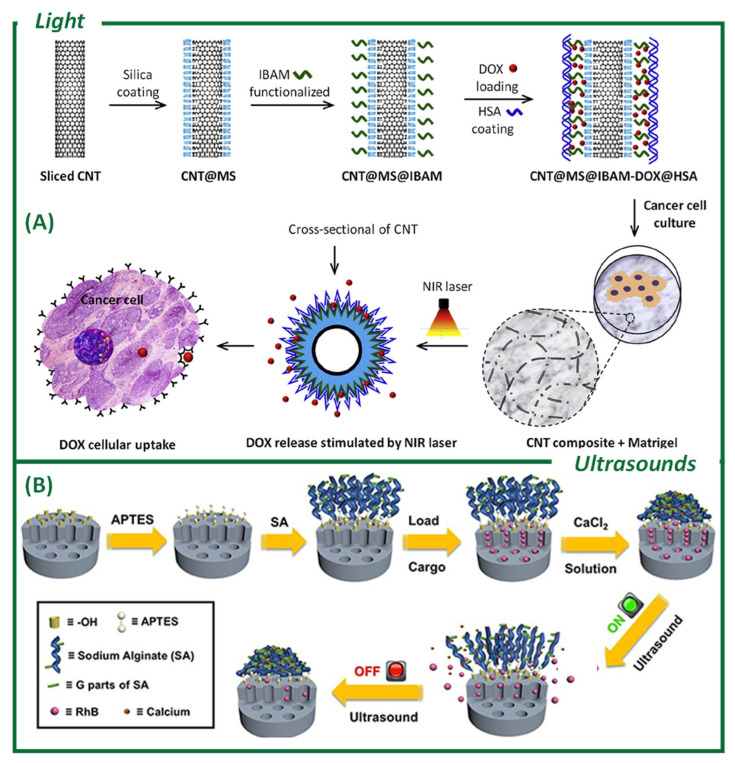
Different examples from the literature of external stimuli-responsive mesoporous silica nanoparticles for anticancer agent release. (**A**) Light: Design of functional CNT@MS nanocomposites to provide phototherapy combined with drug release mediated by NIR laser excitation. Copyright permission from Elsevier (reproduced from Li, B. [142]). (**B**) Ultrasounds: MSNs modified with sodium alginate with carboxyl–calcium (COO^−^-Ca^2+^) coordination bonds in the modified layer, blocking the mesopores. A rapid and significant cargo release is being produced by destroying the bonds under the coordinated stimulation of low-intensity ultrasound (20 kHz) or high-intensity focused ultrasound (HIFU, 1.1 MHz). Reprinted with permission from Frontiers (reproduced from Li, X. [148]).

The use of light for these purposes requires the control of parameters, such as wavelength, intensity, exposure duration, and beam size. There are light-sensitive chromophores that allow for precise reactions [4] and can be coupled to DDSs [139]. Light-mediated mesoporous silica and organosilica nanostructures can be categorized into several groups based on their delivery strategy, including photolysis-responsive nanovectors, photoisomerization-responsive nanovectors, photoredox-responsive nanovectors, and photothermal-responsive upconversion or plasmonic nanovectors. A wide range of the latter, based on mesoporous silica and organosilicon, have been reported as having biomedical applications [140]. Carbon nanotubes (CNTs) have been previously explored as stimuli-responsive tunable absorbers [141]. In a recent study, CNTs were coated with mesoporous silica based on a strategy of combining phototherapy with drug release mediated by near-infrared (NIR) laser excitation (Figure 5A). A very high drug-loading capacity (up to 80% by weight) was ensured by the chemical incorporation of an isobutyramide binder. DOX was used as the drug and its biocompatibility was ensured by adsorbing human serum albumin [142]. Only when the NIR light was directed onto the nanocompound was DOX released, demonstrating the specificity of the strategy. Exploiting NIR light, Tam et al. [143] synthesized LiYbF_4_:Tm^3+^@LiYF_4_ upconverting NPs (UCNPs) coated with mesoporous ultraviolet (UV)-breakable organosilica shells of various thicknesses. The group demonstrated the breakability of the silica framework using an NIR wavelength. Introducing a synthesized light-breakable linker (LB), they coated an NIR-sensitive NP with a breakable mesoporous silica. The nucleus absorbed the NIR light and emitted the UV–visible wavelength which broke the linker. A previous work from the same group from 2020 [144] already demonstrated that it is possible to exploit UV–visible light to break an organic group by synthesizing an organosilica precursor and implementing a silica framework with it. The use of light-responsive SNPs offers promising possibilities for targeted drug delivery, particularly in oncology and phototherapy. However, challenges related to light penetration, material design, and long-term safety must be addressed for its clinical translation. Future work could explore dual-stimuli systems (pH and light) and improved biocompatibility to enhance the therapeutic efficacy.

#### 3.2.2. Ultrasound

An ultrasound (US) refers to pressure waves in a medium with frequencies below 20,000 Hz, which are too low for human auditory perception. Their low absorption by water and tissue allows for non-invasive images with deep penetration and controllable frequencies [4,145]. The use of US offers a non-invasive and precise method for drug delivery while enabling deep-tissue penetration. The aim of US-triggered drug delivery [146] is to enhance the drug concentration selectively at the target site [145]. Shi and colleagues were the first to report on the design and application of silica hybrids for high-intensity focused ultrasound (HIFU) tumor ablation [147]. They utilized Mn-doped hollow MSNs designed for magnetic resonance imaging (MRI) particle tracking in vivo to activate the ablation of tumors under HIFU. Li et al. developed MSNs with reversible responses thanks to the presence of coordination links (COO^−^-Ca^2+^) (Figure 5B) that could be cleaved under low-intensity focused ultrasound (LIFU) (20 kHz) or HIFU (1.1 MHz), releasing the load quickly and significantly [148]. Without the US operating, these links were recovered again and the mesopores and the load release were blocked [149].

## 4. Encapsulation and Delivery of Anticancer Agents: New Approaches

This section highlights the most recent advances in the encapsulation of novel and appealing anticancer cargoes into SNPs. Table 1 summarizes the texture, particle size, pore size, and cargo features of the examples discussed in this section.

### 4.1. Oligonucleotides Loading and Delivery for Therapy

Oligonucleotide-based therapies have emerged as a promising approach in cancer treatment due to their ability to precisely regulate gene expression [150]. These molecules can target oncogenic pathways at the transcriptional or post-transcriptional level, offering a high degree of specificity compared to conventional therapies [180]. Strategies, such as antisense oligonucleotides, small interfering RNA (siRNA), aptamers, and CpG oligodeoxynucleotides, are being actively explored for their potential to inhibit tumor growth, enhance immune responses, and overcome drug resistance [151,152,181]. Therapeutic RNA-based therapies, such as small interfering RNAs (siRNAs) and microRNAs (miRNAs), which aim to silence certain oncogenes and act on gene expression pathways [139,140,141] to halt tumor progression, are promising [151,152,181].

**Figure 6:**
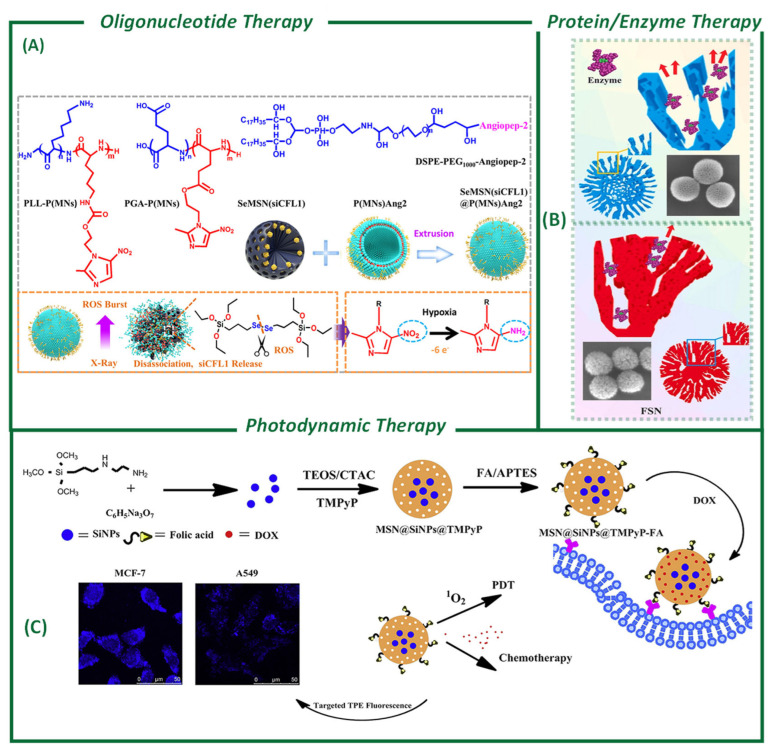
Literature examples of different possible therapies exploiting silica-based nanomaterials as delivery systems. (**A**) Oligonucleotide therapy: Biodegradable selenium-containing mesoporous silica nanocapsule capable of transporting and initiating RNAi by high-energy X-ray irradiation. Reprinted with permission from American Chemical Society (reproduced with permission from Tang, X. [153]). (**B**) Protein/enzyme therapy: Influence of MSN pores (dendritic mesoporous silica nanospheres (DMSNs) with funnel-shaped wide-open pores and fractal silica nanoparticles (FSNs)) on enzyme loading, release, and reusability. Copyright permission from Elsevier (reproduced from Kothalawala, S.G. et al. [158]). (**C**) PDT: Scheme of synthesis of MSN@SiNPs@TMPyP-FA for targeted two-photon-excited fluorescence imaging-guided PDT and chemotherapy. Reprinted with permission from Elsevier (reproduced from Li, S. et al. [173]).

Gu and coworkers described the development of a protective and biodegradable selenium-containing mesoporous silica nanocapsule capable of transporting and initiating RNA interference (RNAi) that inhibits the invasion of recurrent glioblastoma multiforme (rrGBM) (Figure 6A). In detail, it is a nanocarrier that responds to high-energy X-ray irradiation [153]. The results showed high physiological stability, good transcytosis across the blood–brain barrier, and potent accumulation in tumors. After intratumoral administration and low-dose X-ray irradiation, the nanocarrier dissociated, blocking cofilin-1 (a tumor progression protein) and inhibiting glioblastoma cell invasion.

Chen et al. [154] developed silica nanotubes with controllable size distributions conjugated with chitosan to improve CpG oligonucleotide uptake and further enhance immune activation. The size-controlling properties of the silica nanotubes enable shorter nanotubes fabrication, yielding a higher cellular uptake and, consequently, better therapeutic performance. Silica NPs have also been used to simultaneously deliver several antitumor agents to effectively induce an antitumor response. Yantasee and coworkers [182] developed a system for the co-delivery of CpG and STAT3 siRNA using biodegradable silica NPs that induced systemic antitumor immunity, achieving complete tumor regression in melanoma models. Likewise, mesoporous silica vectors carrying tyrosinase-related peptides and TLR agonists were effectively used by Zhu et al. [155] to enhance dendritic cell activation and tumor-specific CD8^+^ T cell responses to tumor immunotherapy. Recent developments also include lipid-coated mesoporous silica NPs for the co-delivery of CpG and TLR7/8 agonists, which modulate the tumor microenvironment by promoting macrophage polarization and T cell infiltration. Additionally, Zheng et al. [183] coated magnetic iron oxide NPs with functionalized mesoporous silica to load CpG, achieving minimal cytotoxicity and efficient cellular internalization. Despite the great potential of this therapeutic strategy, there are some challenges related to stability, enzymatic degradation, and poor cellular uptake that strongly limit ongoing advancements in the field. Thus, NP-assisted delivery may play a critical role in alleviating some of the major obstacles [184]. NP-based delivery systems, including self-assembled NPs [180], lipid NPs, polymeric NPs [156], and inorganic NPs [182], have been exploited as alternative carriers to enhance the therapeutic properties of these nucleotides. This strategy is also currently constrained by safety concerns and their limited efficacy at reaching tumors.

### 4.2. Protein-Based Delivery for Cancer Therapy

The use of protein-based drugs has become a vital approach in the treatment of cancer, metabolic disorders, and immune diseases [157]. Their specificity makes it possible to inhibit tumor cell growth by modulating the TME or stimulating immune responses. Protein therapy has shown less toxicity than chemodrugs and less genotoxicity than gene therapy [185]. However, the delivery of proteins or peptides, shorter aminoacid sequences, to specific tissues or cells is challenging due to their instability during blood circulation, their degradation by enzymes, and immunogenicity. Therefore, delivery systems are being developed that encapsulate, protect, and control the release of proteins or peptides. These systems, based on nanocarriers that respond to pathophysiological stimuli, release proteins on demand in tumor areas or specific subcellular compartments in a controlled manner [185].

The large pore channels of MSNs enable hydrophilic proteins [186] with high molecular weights to be easily encapsulated within them. Du et al. [187] developed biodegradable silica nanocapsules modified on their surface with nuclear localization signal (NLS) peptides. In addition, they coated the NPs with a membrane from cervical–uterine cancer-derived cells (HeLa cell line). These protein and antibody nanocarriers were incorporated into mammalian cells through endocytosis, escaping from endolysosomal vesicles, and ultimately accumulating in the cell nucleus. The in vitro and in vivo results have demonstrated high blood circulation time and the effective inhibition of tumor growth.

Another possibility consists in the use of NPs as transporters of natural enzymes or metabolic interveners. Some of these enzymes can induce tumor cell death, such as ribonuclease A (RNase A), cytochrome C (Cyt C), and granzyme B (GrB). Others regulate the energy metabolism, acidity, and redox balance of tumor cells, such as glucose oxidase (GOx), lactate oxidase (LOx), catalase (CAT), and tyrosinase (TYR). They are very useful in multifunctional strategies where the enzymes are first responsible for altering and sensitizing the sterol methyl transferase (SMT) for greater effectiveness. There are also enzymes that can regulate immune activity, such as kynurenase (KYNase) and adenosine deaminase (ADA). They can be combined with therapies that induce immunogenic cell death (ICD). However, there are risks, such as the involvement of healthy cells, due to their non-specific action [132].

As with proteins, it is possible to covalently attach enzymes to mesopores and investigate the catalytic activity of enzyme–silica complexes. Regarding the pore shape, for example, Kothalawala et al. [158] studied two types of MSNs for phytase and lipase immobilization: fractal (fractal branch-shaped porous structure) and dendritic (dendritic mesochannels with funnel-shaped pores templated by surfactant micelles). The former possessed a higher protein-loading capacity and sustained release behavior, which may be attributed to the complexity of the pore network (Figure 6B). Its enzyme reusability was also higher, highlighting its applicability in catalytic processes.

Protein-based drugs and enzyme therapies offer promising strategies for cancer treatment and metabolic or immune disorders. However, their clinical application faces challenges related to stability, degradation, and immunogenicity, necessitating the development of advanced delivery systems. SNPs have emerged as effective nanocarriers, enabling the encapsulation and controlled release of therapeutic proteins and enzymes. Functionalization strategies, such as cell membrane coatings and nuclear localization signals, have further enhanced their targeting capabilities and therapeutic efficacy.

### 4.3. Photosensitizer-Based Photodynamic Therapy

Photodynamic therapy (PDT) is based on a photosensitizer (PS) placed around the tissue and activated by a light source. The absorbed photon energy creates reactive singlet oxygen, causing oxidative stress and cellular damage, eventually leading to cell death [159]. Selective targeting is crucial, as singlet oxygen is highly reactive and is produced locally by the PS [162]. To promote solubility and overcome aggregation issues, PS molecules are associated with NPs as DDSs [163], leaving the oxygen species to easily diffuse to and from the PS molecule [164].

An example of a durable NP-based DDS that meets the above-mentioned requirements is ultrasmall organic–inorganic hybrid core–shell SNPs stabilized with PEGylated particles. These SNPs are called Cornell prime dots or C-dots, and their size can be controlled on the sub-10 nanometer length scale. One study demonstrated how diagnostic fluorescent C-dots could be converted into therapeutic C-dots by covalently binding the appropriate drug molecules [165]. An example of combining imaging/phototherapy was presented by Li et al. in [173], in which they described the delivery of 5-, 10-, 15-, and 20-tetrakis (1-methyl 4-pyridinio) porphyrin tetra (p-toluenesulfonate) (TMPyP) using MSNs (Figure 6C). The group modified the MSNs on the surface by using folic acid (FA), giving the PS the desired active targeting.

Porphyrins and their derivatives are molecular species widely used in PDT as photosensitizers, thank to their peculiar molecular properties, such as their ability to absorb different light wavelengths. Their encapsulation in a silica delivery system showed their protective [183] effect on these light-sensitive molecules [175]. However, it was reported that when encapsulating proto-porphyrin in SNPs, the administrated dose was almost four times lower than that of a naked PS [160,175]. To overcome the decreasing efficacy of therapy, strategies like the glycosylation of porphyrins by attaching galacto- or gluco-groups [161] can enhance the molecules activity. To enhance the solubility, blood circulation time, and photosensitivity, core–shell porphyrin–silica dots (PSDs) represent a suitable and effective choice. PSDs are ultrasmall NPs with a hydrodynamic diameter of 7 nm, excellent water solubility, and great tumor accumulation; they also display excellent stability in physiological solutions [174].

Different types of organic molecules apart from porphyrins have been used for PDT. Photosensitizers, such as xanthenes, BODYPYs, indocyanine green (ICG), and curcumin, especially work well when combined with chemotherapy (DOX) [166,188,189,190,191,192]. Iodinated BODYPYs are especially interesting in phototherapy, as demonstrated by Zhu et al. in [190], in which the loaded PS in MSNs displayed an IC_50_ = 5 µg/mL in HELA cells. A different plethora of iodinated BODYPYs were investigated by Prieto-Montero et al. [189], and they were able to absorb light at very different wavelengths. Red BODYPYs are relevant for cancer PDT, which have shown a low EC_50_ < 1.0 µM in comparison to free BODYPYs. It is noticeable that all the iodinated BODYPYs developed in [189] possessed relative toxicity even in the dark. Introducing ICG into an MSN system containing DOX and irradiating at 808 nm can decrease the IC_50_ 20 times in comparison when only delivering DOX and 10 times of its free form [188]. Photoactive organic compounds are not only useful for their PS properties in cancer therapy. In fact, as they can absorb and emit light, they can be also exploited as tracing agents to study biodistribution and cellular uptake.

Finally, natural photosensitizers have also been discovered and employed in cancer treatment. An example was given using *Cichorium pumilum* encapsulated in silica nanoparticles in [193]. This natural photosensitizer has shown promising effects in cancer therapy. However, its limited water solubility and low bioavailability have restricted its effectiveness as a photosensitizer for photodynamic therapy. Encapsulating it in a silica framework overcomes the solubility problems, enhancing its efficacy by +157.14%. The effectiveness of PDT depends on precise PS localization and controlled singlet oxygen generation, thereby requiring the development of advanced DDSs [194]. The use of silica nanoparticles, especially MSNs, can efficiently deliver and enhance the effectiveness and solubility of PSs in comparison to their free forms. Still, challenges such as reduced PS bioavailability upon encapsulation remain, prompting the exploration of modifications like glycosylation and core–shell NP engineering.

### 4.4. Metal-Based Catalysts for Cancer Therapy

Metal complexes and metal-based NPs can be delivered and act as catalytic agents able to induce multiple cascade reactions that can alter TMEs [195,196,197,198]. The discovery of cisplatin in the late 1960s [199] led to a significant increase in the use of metallodrugs for cancer treatment [200]. However, the potential of these drugs is limited by their severe side effects, reduced activity over time, and low stability in aqueous solutions [167]. To address these issues, researchers have explored the use of nanostructured materials as alternative vectors for SMT [168,201]. These systems have been shown to be highly effective against cancer cells, and in many cases, the metallodrug-functionalized silica-based nanomaterials act as an entire nanoparticulated therapeutic system. They work as “non-classical” drug delivery nanosystems because they do not release the metal-based drug. The improved cytotoxic action of these systems is due to the high uptake of silica particles by cancer cells, which leads to a different mechanism of cytotoxic action compared to non-encapsulated metallodrugs [168]. As a result, the dynamics of the apoptotic morphological and functional changes are modified when metallodrugs are incorporated into nanostructured silica-based systems [169]. It is also worth mentioning the recent efforts carried out by Unciti-Broceta and Santamaria’s groups related to the development of bimetallic AuPd NPs deposited onto SiO_2_ mesoporous NRs. The SiO_2_ NPs prevented the intracellular deactivation of the metal NPs and enabled the effective activation of paclitaxel using bioorthogonal chemistry [170].

**Figure 7:**
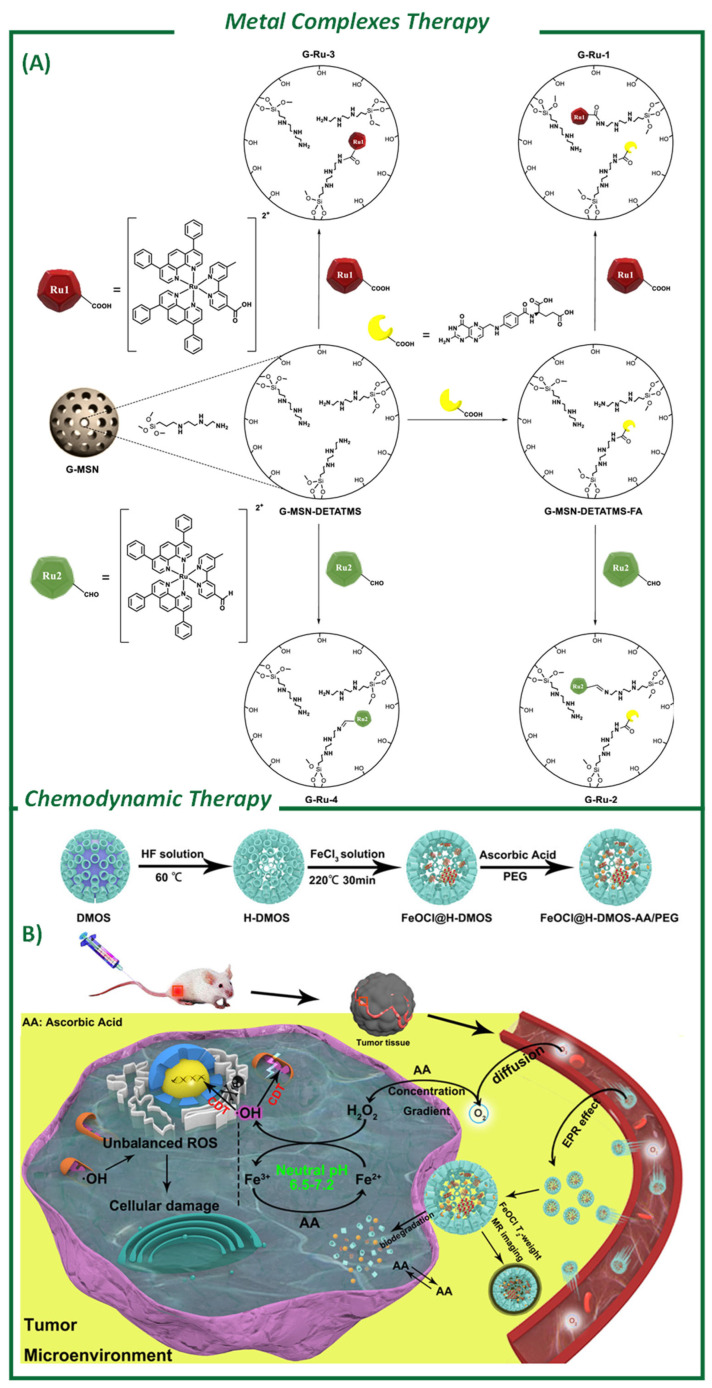
Literature examples of different possible therapies exploiting silica-based nanomaterials as delivery systems. (**A**) Metal complexes therapy: Synthesis of Ru(II) polypyridine complex and folic acid-functionalized MSNs. Copyright permission from American Chemical Society (reproduced with permission from Karges, J. et al. [171]). (**B**) Chemodynamic therapy: Synthesis of FeOCl@H-DMOS-AA/PEG and schematic illustration of proposed CDT strategy. Reprinted with permission from American Chemical Society (reproduced with permission from Li, T. [208]).

Metal complexes, particularly ruthenium (Ru) organic complexes and iridium (Ir) complexes, are being extensively investigated as potential therapeutic agents as PDT PSs due to their ideal photophysical and biological properties. Gómez-Ruiz et al. have published extensive works on Ru complexes [169,171,172] (Figure 7A). The straightforward functionalization of SNPs enables synthesis methods in which a covalent bond is formed between the metal complex and the MSN by amide or imine functional groups on the surface [171,172]. Imine-conjugated NPs were found to have a higher Ru loading and therapeutic efficiency than those conjugated by amide bond formation. It is worth mentioning that Ir complexes are usually highly toxic in free solutions, but the use of NPs has allowed for a safer and more effective utilization of these organic/inorganic photosensitizers compared to traditional organic photosensitizers [202].

Metal-based silica nanomaterials can also be exploited as biosensors [203,204,205,206]. Cancer biomarkers represent a key area within immunoassay research. Kang et al. developed silver-embedded silica nanoparticles (SiO_2_@Ag) for SERS-based immunoassay (SIA) detection of prostate-specific antigens (PSAs), and further explored their potential as multiplex-capable surface-enhanced Raman scattering (SERS) tags for bioimaging applications [207]. Pham et al. developed biosensing systems based on metal-embedded silica in different works. At first, they discovered how a nanosystem based on SiO_2_@Au@Ag and SiO_2_@Au@Au could effectively detect the presence of H_2_O_2_ between 40 and 100 mM [206]. Later, the same group discovered that substituting Au with Pt in a final system made by SiO_2_@Au@Pt could easily expand the range of detection to between 1.0 and 100 mM [203].

#### Chemodynamic Therapy

Considering the compatibility and enhanced features of delivering metal complexes on organosilica NPs, it is easy to imagine exploiting these nanoplatforms also as delivery systems for metallic ultrasmall NPs. As explained in the enzymatic delivery paragraph, new strategies focused on altering the metabolic pathways of tumoral cells are being investigated in cancer therapy [176,209]. The main useful features of these systems are their catalytic and enzyme-like activities [177,210]. These emerging therapies have led to the extensive exploration of nanozymes with enzyme-mimicking catalytic activities in biomedicine. Chemodynamic therapy (CDT), which relies on the Fenton reaction, exploiting overproduced hydrogen peroxide (H_2_O_2_) to generate hydroxyl radicals, has emerged as a potential non-light-based cancer treatment strategy [178]. However, a conventional Fenton reaction only exhibits high efficiency in strongly acidic conditions (pH = 2–4) and in presence of high concentration of H_2_O_2_ [208]. To overcome this potential issue, a straightforward in situ growth approach was employed to confine FeOCl nanosheets within hollow dendritic mesoporous organosilicon (H-DMOS) NPs, resulting in the formation of FeOCl@H-DMOS nanospheres. Ascorbic acid (AA) was absorbed onto the nanosystem to act as a H_2_O_2_ prodrug and facilitate the regeneration of Fenton’s reagent for Fe^2+^ [208] (Figure 7B).

When considering the possibility of exploiting the Fenton rection to generate ROS in tumoral cells, the presence of reductive GSH and high concentrations of Cu-Zinc (Zn) superoxide dismutase could potentially interfere in the process [179,211]. Considering the rationale for chelating the endogenous Cu of Cu-Zn superoxide dismutase followed by performing an intracellular Fenton reaction, Shi and co-workers [212] developed a disulfide bond-containing link poly(acrylic acid) hybrid mesoporous silica nanocomposite for delivering the Cu-chelating molecule N,N,N′,N′-tetrakis(2-pyridinylmethyl)-1,2-ethanediamin (TPEN). The design of this nanomaterial follows the idea of exploiting the S-S bonds to consume the intracellular GSH, releasing the chelating agent, which is further reduced by the GSH acting passively as a GSH oxidative species and active Fenton-reaction initiator.

A novel theranostic nanozyme system (OFeCaSA-V@GA) was developed by modifying biodegradable silicate nanozymes with oxidized sodium alginate (OSA) and gallic acid (GA) [213], enriched with oxygen vacancies (OVs) and Fe–Ca bimetallic active sites. These nanozymes function as efficient photosensitizers for PDT and catalyze ROS generation under 650 nm laser irradiation. The OV structures enhance ROS and CDT by facilitating electron transfer and weakening the H_2_O_2_ bonds. Upon entering tumor cells, the nanozymes degrade in the tumor microenvironment, releasing Ca^2^⁺ and Fe^3^⁺ to induce mitochondrial damage and serve as MRI contrast agents. Additionally, the released OSA and GA self-assemble via metal coordination, enhancing tumor site retention through the assembly/aggregation-induced retention (AIR) effect, supporting their potential in future cancer therapies. CDT faces limitations due to its pH dependency and antioxidant interference; innovative approaches, such as FeOCl@H-DMOS nanospheres and Cu-chelating nanocomposites, could provide promising solutions to enhance its therapeutic efficiency. These advancements have not only improved ROS production in TMEs but have also introduced precise mechanisms to regulate the intracellular redox balance. Recent advances in the use of MSNs for CDT include their transformative potential for the continuous delivery of active Fenton species (Fe^2^⁺) due to their redox-active silica designs, and enzyme-loaded hollow MSNs (GOX, peroxidase) that increase the H_2_O_2_ supply for Fenton chemistry in combination with a glucose-starvation strategy [214]. Strategies that are more complex have used multimodal integration. For instance, under laser irradiation (780 nm), Mn-doped MSNs (Mn-MSNs) showed tumor imaging-enhanced CDT, achieving ROS generation and H_2_O_2_ like those of conventional Fenton catalysts [215].

## 5. Conclusions

Silica NPs have been in the spotlight due to their ability to trap and deliver cargos in tumors. These NPs have shown great potential as drug delivery agents in various therapeutic applications, including CDT, PTT, PDT, siRNA delivery, and RT [106]. However, the main challenges in utilizing Si NPs for drug delivery are related to improving their efficiency in targeting, their stability, and controlled cargo release in the body.

Targeting is a key area of development, with both passive and active targeting strategies being explored. Functionalizing NPs with specific ligands, proteins, or antibodies may enable the efficient recognition of tumor-specific markers in cells [216]. However, these delivery approaches still require refinement to ensure that their non-specific accumulation and macrophage recognition are no longer concerns. Therefore, forward steps in the field will involve the use of membrane-coating technologies, using cancer cell or erythrocyte membranes to improve both bloodstream circulation and internalization efficiency.

Additionally, a fundamental aspect of nanomedicine is the comprehensive understanding of how therapeutic NPs interact with the body. The bio-nano interactions of silica NPs with cell membranes, organs, and critical biological barriers, such as protein corona formation, macrophage recognition, and liver clearance, must be studied to advance knowledge in the field. The size, shape, and surface properties of silica nanoparticles play a crucial role in determining their biological behavior, influencing their ability to traverse biological barriers and their recognition by the immune system [216].

The ongoing research on SNPs development is focusing on improving tumor targeting under different approaches. From a material science perspective, the development of flexible and elastic silica NPs is a promising direction, as these particles may be able to better simulate biological structures, avoid recognition by macrophages, and reach tumors. Additionally, more efficient stimuli-responsive behavior of NPs (e.g., TME-responsive NPs) could provide controlled NPs delivery and in situ cargo release. Furthermore, improving the drug-loading capacity by enhancing the cargo-to-silica ratio is crucial for maximizing the therapeutic potential while limiting the amount of non-native structures. Research on optimizing these factors will lead to more effective and targeted DDSs using SNPs.

Lastly, the clinical translation of silica NPs remains a significant challenge, requiring the development of efficient delivery systems and substantial advancements in the final stages of research. A notable example of successful nanomedicine translation was the development of liposomal nanocarriers, which led to the approval of Doxil, an alternative formulation of DOX currently used in the treatment of certain tumors [217]. Similarly, extensive research has focused on the encapsulation of this molecule within various Si-based nanoplatforms, yielding promising results for cancer treatment. Following this example, researchers should strive to position silica nanocarriers as a viable alternative to both conventional DOX and Doxil, aiming to provide another potentially translatable option with applications in oncology.

## Figures and Tables

**Table 1:** Classification of different therapies based on silicon nanoparticles according to their charge and pore structural characteristics. Missing information is not available in corresponding studies.

Therapy	Cargo	Texture	NP Size (nm)	Pore Size (nm)	Pore Volume (cm^3^/g)	Surface Area (m^2^/g)	References
Oligo-nucleotides	RNAi	Mesoporous	70	6 nm	-	-	[150]
	CpG	Mesoporous	400–1850	2–10	-	-	[151,152,153,154]
Protein-based	HSA-DOX	Mesoporous	100	<1	1.62	708	[155]
	Protein	Capsule	50	-	-	-	[156]
	Phytase/Lipase	Dendritic mesoporous	140	8	≤1	342–399	[157]
Photosensitizer	Silica as Carrier	Core–shell	<10	-	-	-	[158]
	TMPyP	Mesoporous	90	2.1	0.28	223	[159]
	BODYPYs	Mesoporous	50, 80	<6	-	<300	[160,161]
	ICG	Mesoporous	100	2–5	-	83–981	[119]
Carrier	Proto-porphyrin	Non-porous	<10, 60–270	-	-	-	[162,163,164,165]
	*Cichorium pumilum*	Non-porous	25	-	-	-	[166]
Metal-based	Ru	Mesoporous	200	<3	0.7	1045	[167]
	Ir	Mesoporous	150	-	-	-	[168]
	Au@Pt Au@Ag Au@Au	Non-porous	150 Si, 12 Ag, <3 Au	-	-	-	[169,170,171,172]
CDT/PDT	DOX-ICG-DA-HA	Hollow mesoporous	120–270	<3	<2	28	[173,174]
	Curcumin	Mesoporous	<200	<3.5	-	813	[175]
	FeOCl	Hollow dendritic mesoporous	125	10–22	-	29	[176]
	TPEN	Mesoporous	40–50	-	<1	75–450	[177]
	OFeCaSA-V@GA	Mesoporous	130	10–20	<1	-	[178]
	Mn	Mesoporous	80–100	-	-	-	[179]
